# Cause inhabituelle de syndrome du canal tarsien chez une fille de 15 ans: à propos d’un cas

**DOI:** 10.11604/pamj.2022.42.86.35186

**Published:** 2022-06-02

**Authors:** El Hassani Abdelkrim, Hicham Douma, Oussama barchah, Moustapha Elkasseh, Amine Boumaiz, Saad Belkyal, Hanane El Haoury, Mohamed Madhar, Rachid Chafik, Youssef Najeb

**Affiliations:** 1 Service de Traumatologie, Hôpital IBN Tofail de Marrakech, CHU Mohamed VI, Faculté de Médecine et de Pharmacie de Marrakech, Université Cadi Ayyad de Marrakech, Marrakech, Maroc

**Keywords:** Canal tarsien, nerf tibial postérieur, neurofibrome, exérèse, cas clinique, Tarsal tunnel, posterior tibial nerve, neurofibroma, excision, case report

## Abstract

Le syndrome du canal tarsien; c´est l´ensemble des manifestations causées par une compression ou lésion du nerf tibial postérieur (NTP) qui passe dans un canal osteofibreux retromalléolaire appelé le canal tarsien. Dont les étiologies sont très diverses entre autres les tumeurs des nerfs périphériques. Que faut-il savoir suspecter devant ce syndrome résistant au traitement médical et aux infiltrations ? Nous rapportons un cas original d´une fille de 15 ans qui présentait un syndrome de canal tarsien révélant un neurofibrome de NTP, diagnostiqué à tort comme une fascite plantaire et une radiculopathie de S1.

## Abstract (en)

Tarsal tunnel syndrome is a multifaceted condition caused by the compression or lesion of the posterior tibial nerve (PTN) that passes through a retromalleolar osteofibrous canal, the so-called tarsal tunnel. It has multiple etiologies, including peripheral nerve tumors, which must be suspected when this syndrome is resistant to medical treatment and infiltrations. We here report the original case of a 15-year-old girl presenting with tarsal tunnel syndrome revealing neurofibroma of the PTN, misdiagnosed as plantar fasciculitis and S1 radiculopathy.

## Introduction

Les causes de syndrome du canal tarsien, on peut les diviser en causes extrinsèques et d´autres intrinsèques parmi ces dernières on cite les tumeurs des nerfs périphériques [1], représentant 10.2% de toutes les tumeurs du pied et la cheville: schwannome (57%), neurofibrome (29%), les tumeurs malignes(14%) [2]. Ces tumeurs ont une croissance lente expliquant le retard ou manque de leur diagnostic avec une résistance au traitement médical gênant les activités quotidiennes. Nous rapportons ce cas pour alerter les traumato-orthopédistes et les neurochirurgiens d´un autre diagnostic qui devrait être envisagé dans le diagnostic différentiel de la fascite plantaire et les radiculopathies lombaires.

## Patient et observation

**Histoire de la maladie:** fille de 15 ans, étudiante, sans antécédent personnel ou familial, présentant des douleurs chroniques de la cheville droite irradiants vers les orteils et parfois vers la jambe, depuis 1 an, à type de picotement et fourmillement exagérées par la marche et le port des chaussures, soulagées au début par le repos après deviennent très invalidantes avec retentissement sur la vie quotidienne. Sans contexte de traumatisme de la cheville ou du pied. La patiente a consulté plusieurs fois, mais sa symptomatologie était prise à tort comme une fascite plantaire et une radiculopathie, mise sous antalgiques et des infiltrations des anti-inflammatoires non stéroïdiens (AINS) et des corticoïdes (CTC) mais sans amélioration, ce qui a engendré un trouble de sommeil et une dépression chez la fille. Après la patiente a été adressée à notre formation où le diagnostic de neurofibrome de NTP était posé.

**La clinique:** elle a objectivé une petite masse de 3 x 2 cm, solide, immobile, douloureuse à la palpation avec signe de Tinel positif sans aucune faiblesse musculaire ni signes inflammatoires en regard qui peuvent nous orienter vers une ténosynovite du tendon de voisinages ou une arthrite inflammatoire. Dans le cadre de neurofibromatose de Von Recklinghausen; on n´a pas noté des taches café au lit ni d´autres localisations. Avec absence des nodules de Lisch à l´examen ophtalmique.

**Figure 1:**
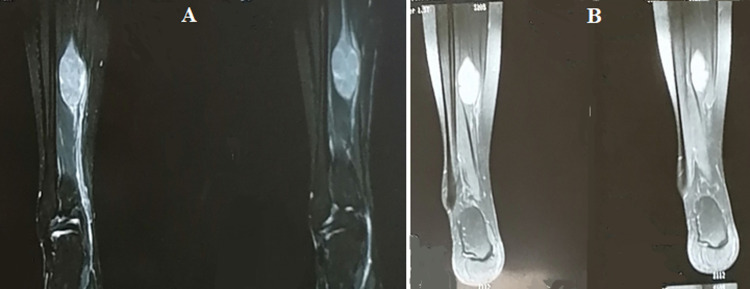
(A, B) imagerie par résonance magnétique préopératoire montrant le neurofibrome du nerf tibial postérieur de notre cas

**L´imagerie:** la radiographie standard de la cheville et du pied était normale. Et pour un bilan lésionnel précis (taille, étendue, profondeur) on a demandé une IRM révélant une masse centrée sur le trajet de NTP d´aspect fusiforme, fasciculée, mesurant 21 x 15 mm étendue sur 40 mm, en hyposignal T1 et hypersignal T2, rehaussée de façon hétérogène après injection de gadolinium, avec portion nerveuse affèrent et efférent, refoulant en dedans le paquet vasculaire restant perméable, entourée par une couronne d´intensité graisseuse: split fat signe (Figure 1).

**Figure 2:**
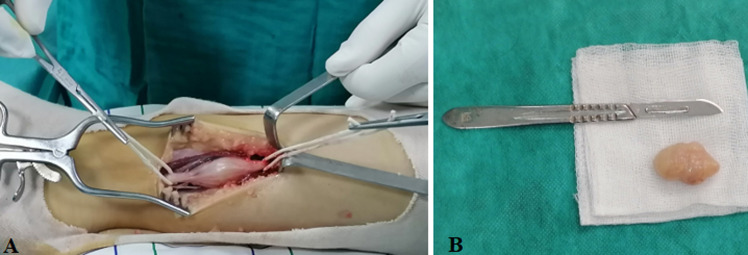
(A, B) image peropératoire de neurofibrome d´aspect blanc jaune, fasciculé sur le trajet du nerf tibial postérieur refoulant le pédicule, avec limites sains isolées dans deux lacs

**Figure 3:**
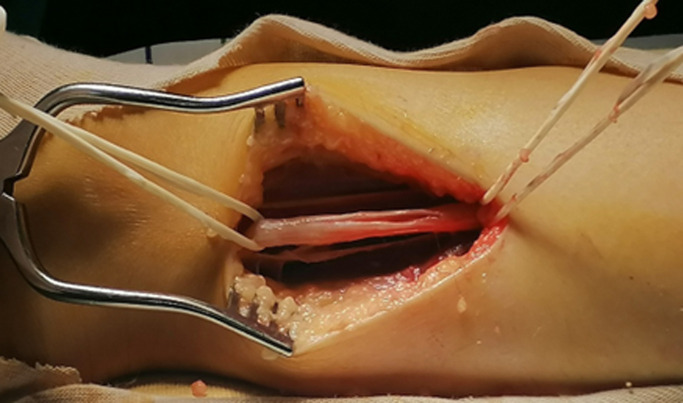
aspect du nerf tibial postérieur après énucléation de la tumeur

**Intervention chirurgicale:** la prise en charge avec les risques ont été discutés avec la fille et sa famille qui ont jugé qu´elle ne pourrait plus vivre avec ces symptômes. Sous rachianesthésie, garrot pneumatique à la racine de membre, incision sur le trajet de la masse après marquage de l’axe des vaisseaux tibiaux avec une soigneuse dissection et hémostase; démontrant une masse jaune. Chamois, encapsulée, d´aspect fasciculé sur le trajet de NTP comme on le voit sur la Figure 2, la masse a été excisée avec respect de continuité de NTP (Figure 3); envoyée après conditionnement pour une étude histologique.

**Figure 4:**
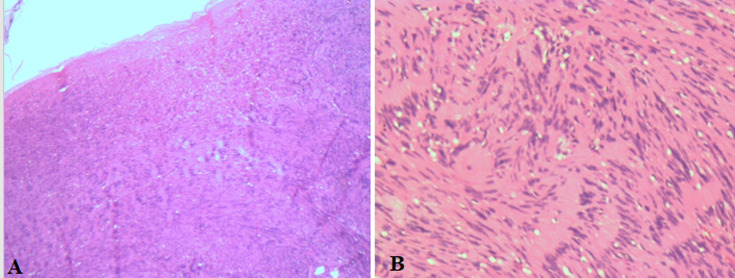
(A, B) lames histologiques montrant un neurofibrome

**Figure 5:**
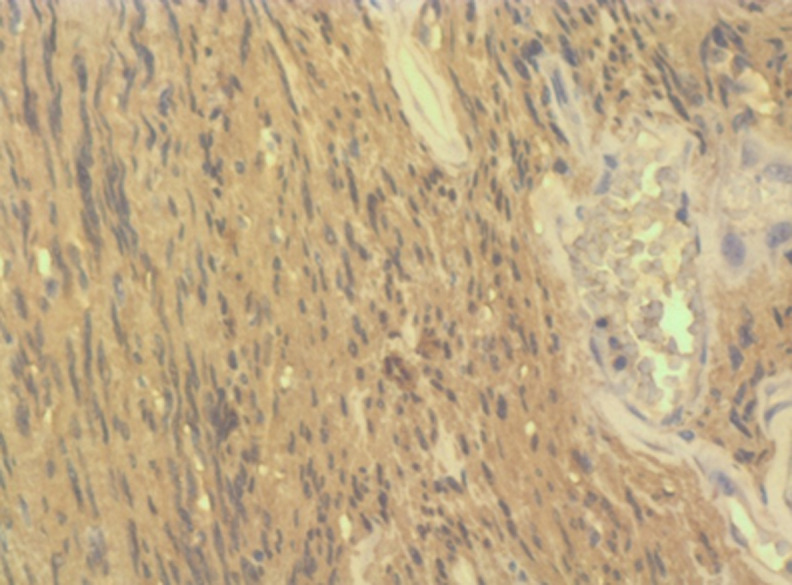
coloration S-100 positive

**Etude histologique:** étude anatomopathologique a confirmé le diagnostic d´un neurofibrome en présences de plusieurs types de cellules schwanniennes, fibroblastiques, mastocytes avec des foyers myxoides et zones ischémiques centrales mais sans signe de malignité (Figure 4); à l´immunohistochimie le marqueur PS-100 était positif (Figure 5).

**Suivi postopératoire:** à trois mois après l´intervention, elle ne rapportait aucune douleur avec signe de Tinel négatif; un an après aucune récidive n´a été décelée en restant toujours indolore avec amélioration de son sommeil et de son humeur.

## Discussion

Le syndrome du canal tarsien est souvent mal diagnostiqué, car peut mimer diverses affections entrainant un retard de diagnostic, comme le rapportait Keck, que ce syndrome est diagnostiqué à tort comme une fascite plantaire [3]; parmi les causes de ce syndrome, il faut penser aux schwannomes et aux neurofibromes du nerf tibial postérieur qui sont des tumeurs bénignes encapsulées. Avec un pic d´incidence entre 3-5^e^ décennie [4], sans prédilection de genre, siégeant souvent au niveau de la tête et du cou, alors que la localisation périphérique est inhabituelle représentant 10% des cas [5], ce qui atteste à la rareté de notre cas. Ces tumeurs surviennent dans le cadre d´une neurofibromatose de Von Recklinghausen. Levi *et al*. ont rapporté que 12 de 34 patients ayant neurofibrome n´avaient aucune association de neurofibromatose [6], faisant l´intérêt de notre cas qui illustre un neurofibrome en dehors de neurofibromatose. On peut expliquer cette dichotomie par [7]: i) si elles surviennent dans le cadre d´une neurofibromatose, une aberration génétique (suppression de gêne suppresseur de tumeur NF-2) est la cause; ii) en dehors de neurofibromatose, un stress environnemental et local (microtraumatisme entrainant un dommage axonal) peut être incriminé. L´examen complémentaire de choix pour ce type des tumeurs est l´IRM permettant de localiser, déterminer la taille de la tumeur et son étendue d´infiltration nerveuse; ce qui est crucial pour une bonne planification préopératoire.

La prise en charge de ces tumeurs consiste à une biopsie excisionnelle et à une neurolyse. Souvent par voie conventionnelle, cependant on peut la faire par voie endoscopique. Avec un bon résultat après exérèse de toute lésion encombrante le canal tarsien [8]. Sung *et al*. ont rapporté 13 cas de syndrome tarsien dus à des lésions occupantes de l'espace, ayant été traités chirurgicalement, avec une amélioration du score des analogues visuels de 6,4 à 2,2 [9], par contre Levi *et al*. ont rapporté que 23,5% des patients présentaient un déficit neurologique après l´excision chirurgicale d´un neurofibrome [10], ce qui n´était pas le cas de notre patiente. Moins de 5% de ces lésions pouvant récidiver, avec risque de transformation maligne faible [5], à moins que le tissu tumoral ne soit incomplètement excisé.

## Conclusion

Le schwannome et le neurofibrome du nerf tibial postérieur doivent être considérés dans le diagnostic différentiel des étiologies de syndrome du canal tarsien. Ces tumeurs peuvent être asymptomatiques alors qu´elles sont petites mais présentaient des neuropathies compressives à mesures qu´elles grossissent. Si elles sont pris en charge correctement elles sont de bon pronostic [11].
